# Observations Concerning *Rhizobium tropici* Bacteroid Phosphorus Stress Response During Symbiosis With *Phaseolus vulgaris*

**DOI:** 10.1111/1758-2229.70220

**Published:** 2025-11-02

**Authors:** Lina M. Botero, Thamir Al‐Niemi, Timothy R. McDermott

**Affiliations:** ^1^ Department of Land Resources and Environmental Sciences Montana State University Bozeman Montana USA

## Abstract

Bacteroid inorganic phosphorus (Pi) metabolism in the *Rhizobium*‐legume symbiosis differs between indeterminate and determinate legume nodules. In contrast to alfalfa bacteroids, bean ( *Phaseolus vulgaris* ) bacteroids exhibit high levels of alkaline phosphatase (AP), the native reporter enzyme for the bacterial Pi stress response. ^14^C and ^32^Pi whole plant labelling techniques were used in conjunction with diagnostic mutants (lacking AP or lacking high affinity Pi transport) to assess the relative importance of the Pi stress response in *Rhizobium tropici* bacteroids during symbiosis. The AP‐ mutant was not defective for symbiosis and did not differ from wildtype bacteroids for Pi acquisition. ^14^C‐CO_2_ feeding to host plants revealed ^14^C‐carbon uptake and accumulation in AP‐ mutant bacteroids, and their nodules were increased relative to wildtype bacteroids, implying that organo‐P compounds may account for meaningful levels of carbon exchange between symbionts. ^32^Pi tracer experiments implied that the high affinity transporter is important to bacteroid Pi acquisition and symbiotic performance in determinate nodules, but that the symbiosome Pi concentration does not meet the capacity of the high affinity transporter. ^32^P tracer work also illustrated that Pi taken up into the nodule does not remain in the nodule, but rather is redistributed to the host.

## Graphical abstract

^14^CO_2_ and ^32^PO_4_ labelling of bean was used with *Rhizobium tropici* mutants. An alkaline phosphatase mutant was not affected for bacteroid P acquisition, though bacteroid and nodule ^14^C‐carbon uptake was altered. The high‐affinity P transporter mutant was defective in nodule P acquisition and symbiotic performance. Plant image by Brgfx/Freepik.

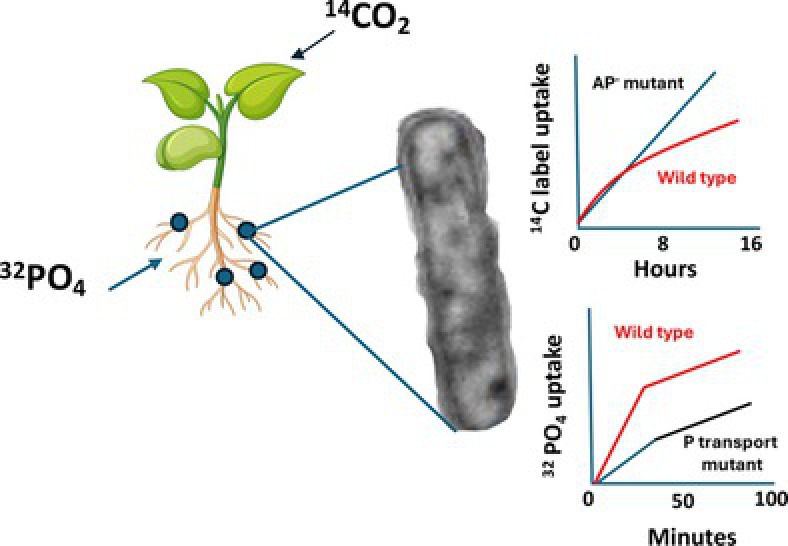

## 1 Introduction

In simplest terms, the *Rhizobium*‐legume mutualistic association is based on the exchange of reduced carbon (C) for reduced nitrogen (N). The host legume photosynthesizes sugars, which are then transferred to the nodule and metabolised to dicarboxylic acids that are then provided to the *Rhizobium* microsymbiont (bacteroid) as an energy source to drive nitrogenase that generates fixed N for the host. This N_2_ fixing symbiosis is important to agriculture worldwide because in many countries legumes are an important dietary protein source and the N can be acquired without fertiliser. However, the agronomic potential of this symbiosis is frequently constrained by available phosphate (Pi) (Graham and Vance 2003; McDermott 1998; Vance 2001; Vance et al. 2003), which often is limiting but also is difficult to provide as a fertiliser. Virtually every aspect of the symbiosis is sensitive to Pi limitation, ranging from infection to nodule function. Alfalfa (Kang et al. 2015; Deng et al. 2001, 1998), soybean (Cassman, Munns, et al. 1981; Cassman, Whitney, et al. 1981; Israel 1987, 1993; Mullen et al. 1988; Pongsakul and Jensen 1991; Singleton et al. 1985), clover (McKay and Djordjevic 1993; Powell 1977), bean (Pereira and Bliss 1987, 1989), chickpea (Itoh 1987) and cowpea (Cassman, Whitney, et al. 1981) show significant positive yield responses to phosphorus fertiliser application, including increased nitrogenase activity and whole plant N concentration, plant dry matter, nodule number and nodule mass. In Pi‐limited legumes, the P concentration in nodules is greater than in other organs and is partially attributed to the very significant host plant investment in mitochondrial and symbiosome membrane synthesis and maintenance (reviewed in Sulieman and Tran 2015).

Plants have evolved highly regulated responses to Pi deficiency. Physiological responses to Pi limitation include increasing the root‐to‐shoot ratio through reduced shoot growth, altered root architecture, increased organic acid exudation and enhanced expression of phosphatases and Pi transporters (Vance 2001; Vance et al. 2003; Raghothama 1999). At the whole plant level, Pi stress responsive genes in the bean legume *Phaseolus vulgaris* have been associated with the upregulation of transport, signalling, carbon metabolism, phenylalanine metabolism and general stress responses. Processes downregulated by Pi stress include starch/sucrose and β‐alanine metabolisms (Hernández et al. 2009; Preiss 1984; Tian et al. 2007).

Pi limitation influences on specific nodule nitrogenase activity varies between studies that have examined chickpea (Itoh 1987), pea (Jakobsen 1985) alfalfa (Deng et al. 2001, 1998), clover (Powell 1977) and soybean (Ribet and Drevon 1995b), with some variation at least partly attributable to differences between legume species, method of plant growth, or method for assessing N_2_ fixation. The host provides photosynthetic sugars to the bacteroids to generate ATP that is essential for N_2_ fixation. Photosynthesis in Pi‐limited legumes is reduced, which then directly reduces photosynthate flow to the root and nodules, and as such negatively affects N_2_ fixation (Fredeen et al. 1990, 1989; Goldstein 1992; Ribet and Drevon 1995a).

In P‐stressed nodules, sugars are channelled into glycolysis and organic acid biosynthesis, most likely through increased phosphoenolpyruvate carboxylase and malate dehydrogenase, suggesting alternative C cycling and P recycling pathways (Jakobsen 1985; Johnson, Allan, et al. 1996; Johnson, Vance, et al. 1996). Soybean nodule nitrogenase activity is linked with Pi availability and nodule energy state (Sa and Israel 1991), with non‐phosphorylating pathways facilitating mitochondrial respiration and Pi recycling involving acid phosphatase activity within the nodule to assist in maintaining maximal ATP for N_2_ fixation under P‐stress growth conditions (Vance 2001; Vance et al. 2003; Hernández et al. 2009; Schulze 2004).

The above highlights of work focusing on the host plant stand in contrast to what we know about bacteroid Pi metabolism. A prior study (Al‐Niemi et al. 1998) has shown that the bacteroid acquires at least some of the Pi taken up by the plant roots or nodules. Clearly, bacteroids have a Pi requirement, but this aspect of the symbiosis remains poorly understood, with little known about how it may influence Pi metabolism and homeostasis at the whole plant system level. Do the plant and bacteroid compete for Pi, and if so, how will the metabolic needs/activities of one influence the other? Alternatively, are their phosphorus metabolisms intertwined in a fashion similar to the exchange of photosynthate from the host for fixed nitrogen from the bacteroids, such that Pi distribution is optimised at the system level in order to maximise symbiotic function?

*P. vulgaris* nodules are strong sinks for Pi taken up either through the nodule surface or transferred to the nodule via vascular tissue from the subtending root (Al‐Niemi et al. 1998). Bacteroid Pi accumulation is significant in these nodules, being particularly enhanced in P‐stressed plants (Al‐Niemi et al. 1998, 1997) and accounting for an appreciable proportion of the Pi taken up by the nodule. In both *Glycine max* and *Medicago truncatula* nodules, a high‐affinity P transporter facilitates P transfer from vascular tissue to the nodule and is necessary for the functioning of the soybean nodule under low Pi conditions (Qin et al. 2012; Nguyen et al. 2021). Bacteroid Pi metabolism in indeterminate nodules (e.g., *Medicago sativa* and *Medicago truncatula* ) differs from determinate nodules (e.g., *P. vulgaris* ). Current evidence strongly suggests that bacteroids in indeterminate nodules take up Pi via their low‐affinity P transporter in a nodule/symbiosome environment that is relatively high in Pi (Nguyen et al. 2021; Yuan et al. 2006), the latter being implied from the lack of alkaline phosphatase (AP) activity in alfalfa bacteroids. This is in contrast to *P. vulgaris* (bean) bacteroids where AP is highly expressed regardless of the Pi nutrition status of the host plant (Al‐Niemi et al. 1998). AP expression is important because it is a native reporter enzyme for the Pi stress response in bacteria (Wanner 1996; Santos‐Beneit 2015), and as such implies that Pi availability in the nodule and/or symbiosome environment differs between indeterminate and determinate nodules. This has many possible implications that extend beyond the function of AP per se or for Pi acquisition. Work with *Escherichia coli* showed that the Pi stress response in Gram‐negative bacteria is truly global in nature, wherein expression of hundreds of genes is influenced by Pi limitation (VanBogelen et al. 1996; Metcalf et al. 1990). Subsequent work with *Sinorhizobium meliloti* revealed similarly comprehensive changes in gene expression (Summers et al. 1998; Krol and Becker 2004), spanning all aspects of cell metabolism and physiology. Consequently, bacteroid AP expression signals that bacteroid cell metabolism is changed significantly because of Pi limitation and presumably could influence symbiotic performance.

We have been studying the Pi stress response in the bean symbiont *Rhizobium tropici* strain CIAT 899 as a way of examining how much and what form of phosphorus the bean host plant provides to the bacteroids in the nodule (i.e., Pi or some form of organo‐P). In broth‐cultured cells, *R. tropici* CIAT 899 AP expression is typical of that observed in many bacteria, up‐regulating when media Pi levels decrease to approximately 0.5 μM (Al‐Niemi et al. 1997; Botero et al. 2000). As noted above, high levels of AP in *R. tropici* bacteroids in nodules of bean plants grown with high Pi nutrient solution (Al‐Niemi et al. 1997) suggest that even though Pi levels in the nutrient solution surrounding the nodule are quite high, the bacteroids nevertheless experience a P‐limiting environment within the symbiosome, resulting in the up‐regulation of the phosphate stress response (Pho regulon). Interestingly, however, we also observed *R. tropici* bacteroid AP levels decline as the symbiosis matures (Al‐Niemi et al. 1997), suggesting the possibility that the host may increase Pi allocation to the bacteroid at later stages of the symbiosis, resulting in down regulation of AP. To more thoroughly examine the relative importance of bacteroid Pi stress response in the functioning of the bean symbiosis, we conducted studies that combined the use of selected *R. tropici* mutants with plant labelling techniques to study the relative importance of the high affinity Pi transporter and AP in *R. tropici* bacteroids for symbiotic function.

## 2 Experimental Procedures

### 2.1 Growth Media, Bacterial Strains and Mutant Isolation

Using methodology we have used previously (Summers et al. 1998; Simon et al. 1983), we generated mutants of *R. tropici* strain CIAT 899 that lack AP. Briefly, this includes the conjugation of Tn*5* (encodes kanamycin resistance) from *Escherichia coli* S17‐1 (Simon et al. 1983) and screening *R. tropici* transconjugants for the targeted AP phenotype. The *phoA*::Tn*5* mutant lacking AP (designated herein as AP‐) was isolated as a Kan^R^ white colony among thousands of blue colonies on low phosphate minimal mannitol agar (Summers et al. 1998) modified to include kanamycin (50 mg L^−1^) and 5‐bromo‐4‐chloro‐3‐indolyl phosphate (BCIP, 40 mg L^−1^) as a chromogenic phosphatase substrate. To identify the interrupted gene, the transposase arm of Tn*5* along with adjacent chromosomal DNA was cloned from total DNA extracts of the mutant using arbitrary priming PCR techniques we have previously described (Kashyap et al. 2006). The Tn5‐genomic DNA junction was sequenced and then BLAST searched, which yielded a sequence that was 100% identical to *phoA*, which is the encoding gene for AP and that was previously published in the genome sequence for strain CIAT 899 (Ormeño‐Orrillo et al. 2012) (Genbank reference sequence NC_020062.1). We previously described the isolation and characterisation of *R. tropici* strain CAP45, which lacks the high affinity Pi transporter and that expresses AP constitutively (Botero et al. 2000). Relative changes in bacteroid AP activity over the course of the symbiosis were assayed by isolating the bacteroids from sampled nodules using methods previously described (Al‐Niemi et al. 1997) and then recording the hydrolysis of the synthetic AP substrate *p*‐nitrophenyl phosphate as Δ Ab_405_ per minute (Smart et al. 1984) normalised for bacteroid dry matter which was derived from pre‐established standard curves that correlated bacteroid optical density to bacteroid cell dry matter (Botero et al. 2000).

### 2.2 Plant Inoculation and Cultivation

Axenic *P. vulgaris* (cultivar Viva Pink) seedlings were prepared by surface sterilising the seeds and germinating on sterile nutrient agar using previously described methods (McDermott and Kahn 1992). Seedlings showing no signs of microbial growth were aseptically transferred to sterile plastic growth pouches (McDermott et al. 1991) and inoculated with either *R. tropici* wildtype CIAT899 or one of the mutants described above. Plants (two per growth pouch) were cultured with sterile Pi‐sufficient nutrient solution (Al‐Niemi et al. 1997), but were also alternately replaced with sterile distilled water to avoid salt accumulation. Plants were grown in a growth chamber providing a photon flux density of 80 μmol s^−l^ m^−2^ during a 16 h photoperiod at 25°C. Four replicate growth pouches were used per treatment (total of eight plants). Data were subjected to analysis of variance (ANOVA), with means compared using Tukey's HSD and significance established using *p*‐values < 0.05.

### 2.3 Radioisotope Labelling

^32^P labelling experiments were conducted using the same methods we have described previously to examine bacteroid Pi acquisition in vivo (Al‐Niemi et al. 1998, 1997). Briefly, this involved the transfer of growth pouch‐grown plants to beakers containing half‐strength nutrient solution containing 325 μM [^32^P]KH_2_PO_4_ (specific activity of 0.45 μCi. μmol^−1^ PO_4_). Plants were suspended such that the entire root system was submerged, with nodules sampled after varying periods of exposure. Nodule sampling and washing, as well as bacteroid extraction, washing and determination of ^32^P content were also previously described (Al‐Niemi et al. 1998, 1997).

^14^C labelling experiments used protocols modified from those first described by Reibach and Streeter (1983). A single leaf on each 32‐day‐old plant (three replicate plants per bacterial inoculation treatment) was sealed in a plastic bag and exposed to 150 μCi ^14^CO_2_ for 4 h (^14^CO_2_ liberated by adding 2 M HCl to NaH ^14^CO_3_) and followed by a chase period where nodule and bacteroid ^14^C content were measured after 0, 4 and 16 h. Whole nodule and bacteroid ^14^C contents were measured by overnight digestion in 1.0 mL Soluene, followed by dilution with 19 mL Hionicfluor (Packard), and then ^14^C was measured using scintillation analysis.

## 3 Results

### 3.1 Symbiotic Phenotypes of Mutants Included in This Study

**FIGURE 1:**
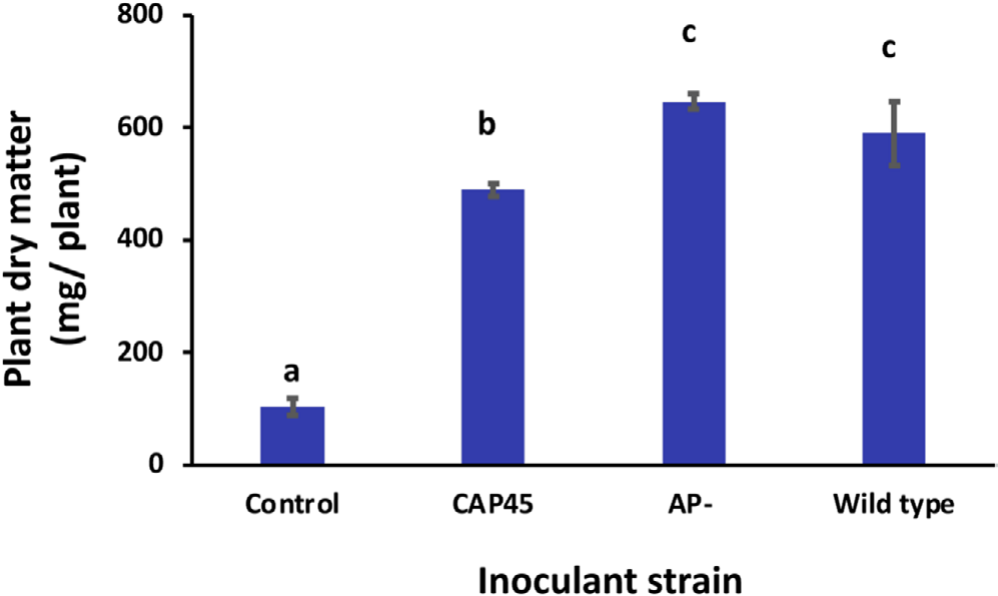
Symbiotic phenotype of *Rhizobium tropici* wild type strain CIAT899, the alkaline phosphatase mutant (AP‐) and the phosphate transport mutant (CAP45). Symbiotic phenotype after 30 days was evaluated based on plant dry matter production directly related to biological nitrogen fixation by the different strains used as inoculants in the treatments. Control plants were not inoculated and did not have nodules. Data are the means ± SE of four replicate growth pouches. Bars with the same alphabetic letter are not statistically different when testing at *p*‐value < 0.05.

The lack of bacteroid AP did not translate into any obvious symbiotic defect (Figure 1). Dry matter for plants nodulated by the AP‐ mutant was not statistically different than plants nodulated by the wildtype strain CIAT 899. However, plants nodulated by the CAP45 mutant (lacks high affinity Pi transport) displayed significantly reduced growth (Figure 1), although still greater than the non‐nodulated control plants (*p*‐value < 0.05) and nodules were pink. Bacteroids isolated from nodules formed by the AP‐ and CAP45 mutant retained their AP activity phenotype as well as their antibiotic marker (results not shown), illustrating that the mutations were stable upon passage through symbiosis.

### 3.2 Bacteroid P Acquisition

**FIGURE 2:**
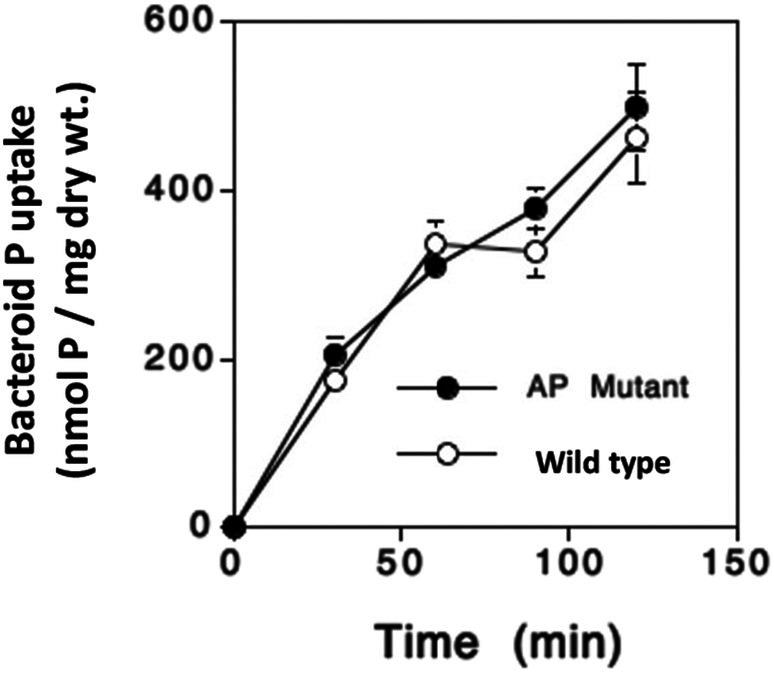
Lack of alkaline phosphatase (AP‐ mutant) appears to have no effect on bacteroid P acquisition in the nodule. P is calculated assuming all P taken up is ^32^Pi. Bacteroids were harvested and purified from nodules of 35‐day‐old plants at the different times indicated (minutes). Data represent and are the means ± SE of bacteroid samples taken from four replicate growth pouches.

**FIGURE 3:**
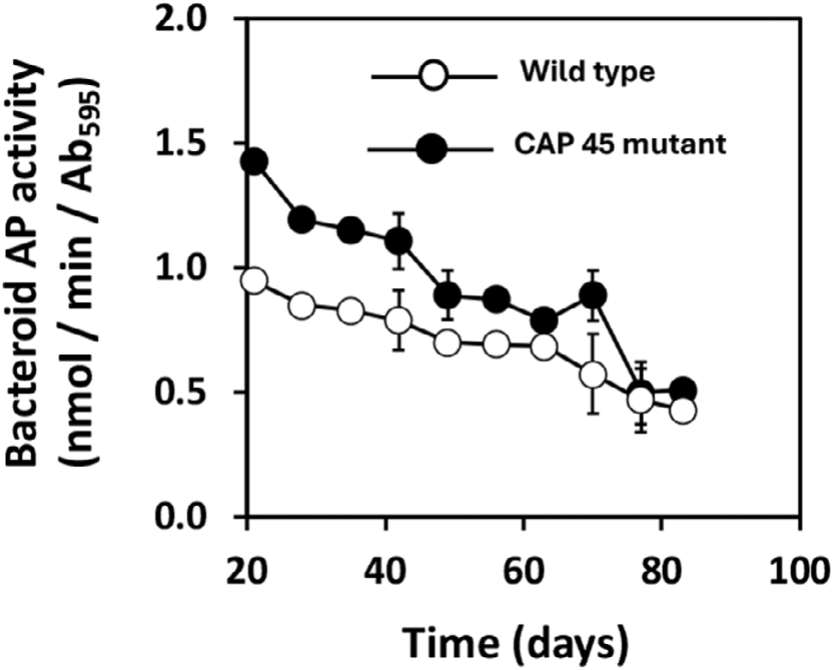
Bacteroid AP activity in wild type strain *Rhizobium tropici* CIAT 899 and the CAP 45 regulatory mutant over the course of the plant growth cycle. Bacteroids were harvested from nodules at the time points indicated (days of symbiosis, X‐axis) and assayed for alkaline phosphatase (AP) activity. AP was normalised based on cell density of the bacteroid preparation that was calibrated to cell protein content. Data are the mean ± SE of purified bacteroid samples (normalised to optical density) from three replicate plants taken at each sampling time point. Error bars, where visible, represent one SE of the mean.

The AP‐ mutation did not influence bacteroid P acquisition (Figure 2). After a 120 min labelling period, ^32^Pi acquisition totaled 499 ± 51 and 463 ± 55 nmol P·g‐bacteroid dry wt^−1^ for AP‐ and CIAT899, respectively (calculations based on specific activity of the label and assumed all P acquired was as Pi). Similar results were obtained in a second independent experiment (results not shown). Because we previously observed wild type *R. tropici* CIAT 899 bacteroid AP activity to decline during the life span of the symbiosis (Al‐Niemi et al. 1997), it was of interest to determine whether this was due to down‐regulation of the encoding gene (*phoA*) during later stages of symbiosis (e.g., host plant increasing Pi allocation to bacteroids) or if it could be due to loss of bacteroid structural integrity that resulted in loss of this periplasm‐based enzyme during bacteroid purification. The CAP45 mutant strain overexpresses AP constitutively and its expression is not linked to Pi availability (Botero et al. 2000), making it an ideal contrast to the wild type strain CIAT899. Because of AP overexpression in CAP45, the observed initial greater AP activity in the CAP45 mutant bacteroids relative to the CIAT 899 wildtype strain was expected (Figure 3). Over the course of the symbiosis, AP activity decreased in bacteroids of both strains, confirming a single time point analysis of our prior work (Al‐Niemi et al. 1997) but also suggesting that decreasing bacteroid AP activity was not due to down‐regulation of AP in the bacteroids.

**FIGURE 4:**
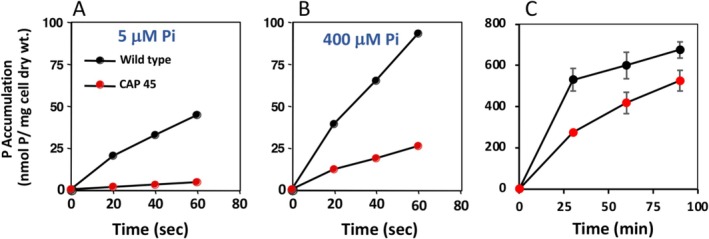
Pi uptake profiles of duplicate mid‐log phase cultures of wild type CIAT 899 and the CAP 45 mutant. (A) ^32^Pi transport profiles of wild type CIAT 899 and CAP 45 in pure culture under Pi conditions where only the high affinity transporter would be saturated (5 mM Pi) or (B) Pi saturating conditions where both the high affinity and low affinity Pi transporters would be saturated (400 mM Pi). Error bars in panels A and B are hidden by the data symbols. (C) Bacteroid ^32^P label accumulation *in planta* and calculated assuming all P is taken up as ^32^Pi. Data are the means of bacteroid samples taken from nodules of four replicate plants (3 days). Error bars indicate ± one SE.

The CAP45 mutant also lacks the expression of the high‐affinity phosphate transporter (Botero et al. 2000) and so it was of interest to assess if the Pi transport phenotype was evident in the nodule environment and to probe the bean symbiosome environment in the context of Pi availability in the bean symbiosome. We previously characterised the kinetics of the two Pi transport systems in *R. tropici* wildtype CIAT 899 (Botero et al. 2000). *K*_m_ values for the CIAT 899 Pi transport systems were estimated to be 34 ± 3 μM Pi and 0.45 ± 0.01 μM P_i_ for low and high‐affinity transporters, respectively (Botero et al. 2000). Figure 4 illustrates the Pi uptake profiles of the cultured wildtype and CAP45 mutant under conditions of defined Pi availability meant to bracket any reasonable estimate of Pi concentrations in the symbiosome. Specifically, Pi transport in cultures was assayed with 5 μM Pi (i.e., 10‐fold the *K*_m_ of the high‐affinity transporter), where only the high‐affinity transporter would be saturated (Figure 4A), and also at 400 μM Pi (10 × low‐affinity transporter *K*_m_) where both systems would be saturated (Figure 4B). Under these conditions, wildtype CIAT 899 Pi uptake exceeded CAP45 by nine‐fold and four‐fold under low and high Pi growth conditions, respectively, and thus was consistent with the high‐affinity P transport defect of the CAP45 mutant (Botero et al. 2000). Additional experiments then followed where nodulated roots were fed ^32^Pi to examine bacteroid in vivo P label acquisition by the CIAT 899 wildtype and CAP45 strains. The same general Pi uptake trend was observed, although the wild‐type bacteroid ^32^P label uptake only exceeded the CAP45 mutant by roughly two‐fold after 30 min of uptake and then 1.3‐fold after 90 min of label uptake (Figure 4C). The reduced differential for ^32^Pi uptake between the wildtype and mutant bacteroids suggests that in the symbiosome environment, the bacteroid high‐affinity P transporter is well under saturated.

**FIGURE 5:**
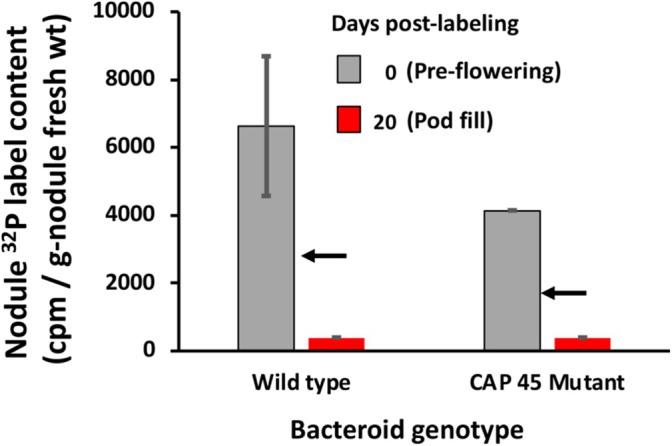
Bacteroid ^32^P label acquisition influences total nodule ^32^P levels and evidence of P cycling out of nodule during the 20 days post‐labelling period coinciding with pod fill. Grey bar represents the ^32^P label in nodules harvested immediately after the exposure period (plants 30 days old), whereas the red bar represents ^32^P label content at 20 days post labelling. Arrows indicate ^32^P radioactivity (^32^P half‐life = 14.3 days) if the decrease in ^32^P cpm was due solely to radioactive decay. Data are from nodules of three plants for each treatment. Where visible, error bar = 1 SE.

Our prior efforts showed that the bean nodule can act as a strong Pi sink (Al‐Niemi et al. 1998), and so additional experiments were conducted to assess the importance of bacteroid Pi transport for total nodule Pi sink strength and to also assess the fate of Pi after it enters the nodule. More specifically, is Pi entry into the nodule unidirectional, or will it exit the nodule to begin circulating systemically within the plant? Plants nodulated by CIAT 899 wild type and the CAP45 mutant were exposed to ^32^Pi as in the above bacteroid Pi uptake experiments, and then a subset of nodules were selected, briefly washed to remove surface‐associated label, and then homogenised and assayed for total ^32^Pi. Approximately 40% less ^32^Pi label accumulated in nodules formed by the CAP45 mutant relative to wild type nodules (Figure 5), and this reflects the importance of bacteroid high affinity P transport activity for total nodule Pi uptake. Importantly, however, after an additional 20 days, remaining ^32^P in the nodules was not affected by the bacteroid P transport, decreasing 89%–93% of that registered on the day of labelling (Figure 5). This decrease cannot all be accounted for by radioactive decay (^32^Pi has a 14.3‐day half‐life, indicated by arrows in Figure 5).

**FIGURE 6:**
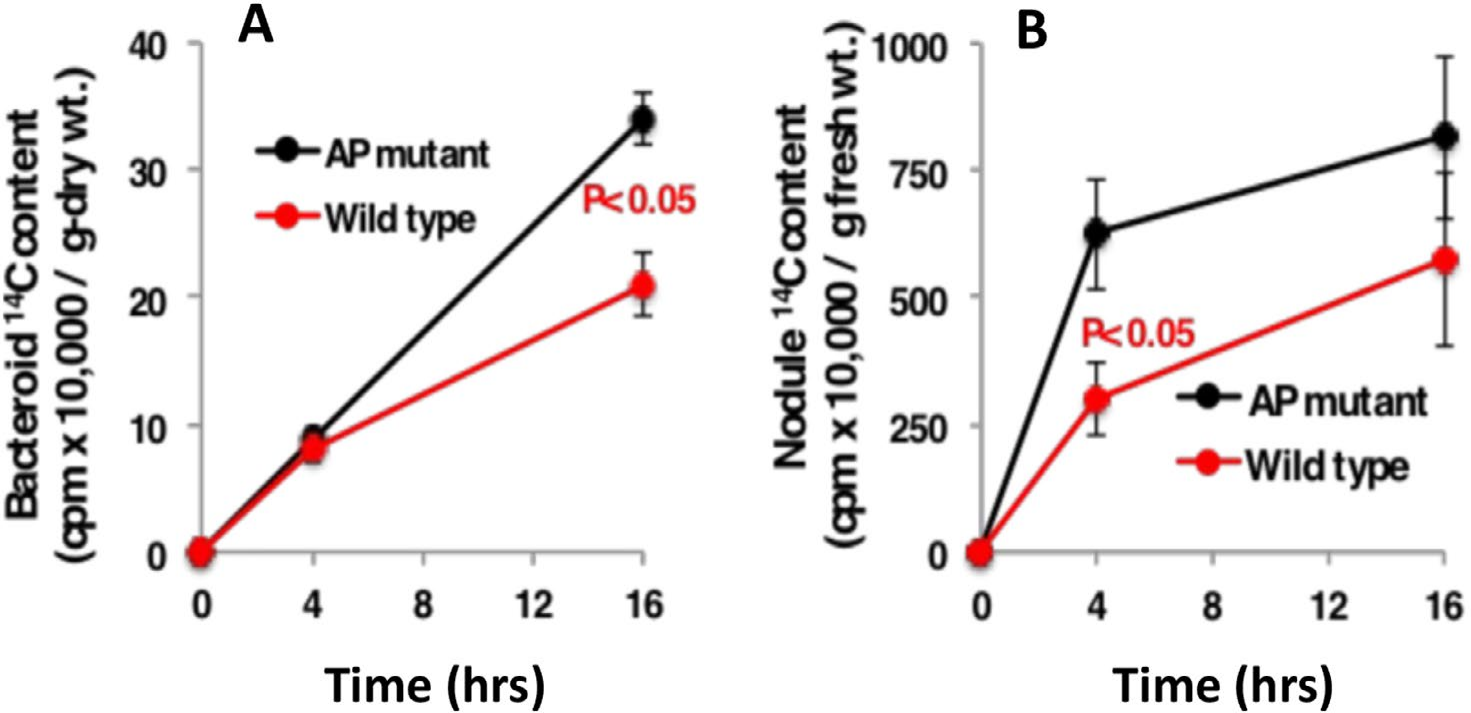
Bean bacteroid (A) and nodule (B) ^14^C acquisition profiles influenced by bacteroid alkaline phosphatase activity. Shoots of growth pouch plants (36 days) grown in high Pi nutrient solution (750 mM Pi) were exposed to ^14^C‐CO_2_ for 4 h, followed by a chase period where samples were taken at 4 and 16 h. Data are the means ± SE of bacteroid samples taken from four replicate plants.

Finally, because bacterial uptake of some carbon compounds is influenced by phosphorylation state and specificity of transporters, additional labelling experiments were conducted to determine if loss of bacteroid AP function could influence bacteroid carbon acquisition in the nodule. After a 4 h chase period, the ^14^C label content in the AP‐ mutant bacteroids was not different from CIAT 899 wild type bacteroids (Figure 6). At the 16 h sampling point, however, there was a statistically significant difference, with the AP‐ mutant bacteroids accumulating significantly more label (Figure 6A). As well, whole nodule ^14^C acquisition profiles of nodules formed by the AP‐ mutant were significantly greater than the wild type nodules at the 4 h chase period (*p*‐value < 0.05) and continued to trend greater at 16 h, although differences were not statistically significant (Figure 6B).

## 4 Discussion

Major elements of the Pi stress response in bacteria include upregulation of AP and the high‐affinity Pi transporter (Wanner 1996). Evidence of upregulation of the Pi stress response in legume nodule bacteroids was first documented in *R. tropici* bacteroids, wherein very significant bacteroid AP expression was observed regardless of the Pi nutrition status of the host bean plant (Al‐Niemi et al. 1997). This suggests that the bacteroid in the bean symbiosis is provided very low levels of Pi. More recently, the expression of the high‐affinity P transport system was documented in bacteroids in nodules of bean, soybean and pigeonpea (Hu et al. 2018). Together, these observations are consistent with the view that the symbiosome environment for bacteroids in determinate nodules contains low levels of P that then results in the induction of the Pi stress response. Based on studies with the relevant pure culture rhizobia (Al‐Niemi et al. 1997; Yuan et al. 2006; Botero et al. 2000) and the results of the current study, this suggests the symbiosome in determinate nodules contains < ~0.5 μM P (Figure 4) and further suggests the symbiosome membrane in determinate nodules may be less efficient in translocating Pi to the bacteroids. This contrasts with bacteroid P metabolism in indeterminate nodules (e.g., alfalfa), where it has been concluded that bacteroids are provided sufficient Pi by specific host transporters (Nguyen et al. 2021) such that the bacteroid Pi stress response is not induced (Nguyen et al. 2021; Yuan et al. 2006; Hu et al. 2018).

In the current study, the negative symbiotic phenotype observed for the *R. tropici* Pi transport mutant (CAP45, Figure 1) is consistent with Hu et al. (2018) who showed that the bacteroid high affinity Pi transporter is required for effective symbiosis in other determinate nodule symbioses. ^32^P uptake in wild‐type CIAT 899 bacteroids exceeded that of the CAP45 mutant (Figure 4C), but the increase was small relative to what might be predicted based on differential transport assays of these strains in pure culture where Pi levels were manipulated to demonstrate potential differences when one or both transport systems are fully engaged (Figure 4A,B). This suggests that Pi in the symbiosome space occurs at very low concentrations. A more direct approach for estimating the actual symbiosome Pi concentration derives from prior work (Al‐Niemi et al. 1997) which showed that induction of the CIAT 899 *phoA* (AP encoding gene) occurs at a Pi ~0.5 μM. As such, CIAT 899 bacteroid AP expression argues that the Pi levels in the symbiosome space would be at, or below, this same induction level (~0.5 μM Pi). This actually matches the Km of the high affinity Pi transporter in CIAT 899 (0.45 ± 0.01 μM P_i_, Botero et al. 2000), implying that the prevailing Pi in the symbiosome space is at or below half‐saturation for this transporter. This also suggests that symbiosome Pi is ultimately constrained by the lack of efficient Pi transporters in the symbiosome membrane.

P uptake by bean nodules is quite robust, and in hydroponic environments, these nodules can strongly compete against root tissue for available Pi (Al‐Niemi et al. 1998). The nodule Pi uptake data in the current study extends this earlier observation, showing similar rates of ^32^Pi uptake and that total nodule P accumulation is influenced by the bacteroid Pi transport activity (Figure 5). Regarding P equilibrium and homeostasis in this symbiosis, these experiments illustrate two key, novel observations thus far undocumented for any symbiosis, determinate or indeterminate. First, while some of the label detected in nodules was no doubt symplastic, the fact that significant label penetrated to the bacteroids (Figures 2 and 4) clearly illustrates that Pi movement within the nodule is rapid and complete. Second, and perhaps even more importantly, labelled P (in whatever form) clearly effluxed from the bacteroids/nodules, indicating P allocation within the symbiosis is dynamic. Mediated by PHO1 (Hamburger et al. 2002), Pi will move from the plant cell via efflux (Mimura 1999), and then transported symplastically to the xylem for movement to the shoot. It is unclear how and in what form the P exits the bacteroid and nodule, but P release to the host plant was evident during the 20 days following initial labelling (Figure 5). This time frame coincided with the host plant transitioning to intense reproductive activity; that is, seed formation and pod fill. It is well established that during seed formation, it is normal for a plant to redirect nutrients to the forming seed as a means of maximising viability of the plant's offspring. Bacteroid involvement in nodule Pi accumulation (at least ~60% of nodule Pi accumulation, Figure 4C) and then followed by significant loss of ^32^P label (beyond radioactive decay, Figure 5) is evidence that bacteroids in bean nodules play a significant role in establishing P equilibrium in the symbiosis. Thus far, P homeostasis models for this symbiosis have not included bacteroids (Sulieman and Tran 2015).

Previous studies showed that bean bacteroid AP activity declined as the symbiosis matures (Al‐Niemi et al. 1997), and thus we were interested in obtaining a better understanding of this nodule age‐related phenomenon. The mutant CAP45 constitutively expresses AP (Botero et al. 2000) and thus was ideal to address this question, and showed the decline in bean bacteroid AP activity is the same for the wild type CIAT 899 and the CAP45 mutant (Figure 3). If down‐regulation of the encoding gene (*phoA*) is involved in bacteroid AP decline, then AP in CAP45 would not be expected to decrease (i.e., its AP profile would remain more or less flat). This did not happen (Figure 3), leading to the interpretation here that the decline in bacteroid AP activity is not a result of down‐regulation of the encoding gene in the bacteroids, but rather a technical issue concerning bacteroid fragility and senescence at later stages of symbiosis that leads to loss of the enzyme during bacteroid collection from nodule homogenates. Bacteroids in indeterminate nodules would not be expected to have similar profiles because of how the nodule is separated into the nodule meristem/invasion tissue, actively N_2_‐fixing and senescent zones. Bacteroids in indeterminate nodules undergo significant morphology changes when they leave the infection thread and become active in fixing nitrogen; that is, they become swollen and very fragile. Only bacteroids in the persistent infection threads would be expected to remain viable and structurally sound, whereas differentiated bacteroids in the fixing zone and senescent bacteroids would readily lyse in nodule homogenates and attempted bacteroid purification.

Lack of a negative symbiotic phenotype of the *R. tropici* AP‐ mutant (Figure 1) implies that this enzyme is not required for bean bacteroids to be fully functional in symbiosis. Indeed, plant dry matter generated by plants nodulated by the AP‐ mutant trended to be greater (Figure 1). Lack of bacteroid AP activity also does not appear to influence bacteroid Pi acquisition (Figure 2), which indicates that the bean symbiosome does not contain appreciable organo‐P compounds that could contribute significantly to bacteroid P uptake—at least relative to levels of Pi taken in via Pi transporters. By contrast, the bacteroid AP enzyme does influence bacteroid and nodule carbon acquisition as judged by a ~75% increase in ^14^C label accumulation by the AP‐ mutant bacteroids relative to CIAT 899 (Figure 6) and suggests that phosphorylated carbon compounds that are sensitive to AP are available to the bacteroid in the bean symbiosome. This would require relevant organo‐P compounds as well as matching transporters. Documented bean nodule phosphorylated metabolites include fructose‐6‐phosphate, glucose‐6‐phosphate and 3‐phosphoglycerate (Hernández et al. 2009). The CIAT 899 genome carries the Ugp (uptake of glycerol‐3‐phosphate) Glp (glycerol‐3‐phosphate transporter), but these are specific for glycerol 3‐phosphate and will not facilitate transport of any of the above phosphorylated metabolites. *Rhizobium leguminosarum* biovar *phaseoli* bacteroids have been shown to express UgpABCE in bean nodules as well as a large Glp operon (Green et al. 2019), which could enhance C uptake if: (1) glycerol‐3‐P is transported across the symbiosome membrane and made available to the bacteroids; and (2) the bacteroid does not express a phosphatase enzyme that would otherwise eliminate glycerol‐3‐P as a substrate for Ugp or Glp transporters.

If an organo‐P compound is taken up, then one might expect increased C to be accompanied by increased P. The apparent disconnect between ^14^C accumulation and ^32^P in the AP‐ mutant bacteroids might potentially derive from the expected distinct differences in how ^32^Pi and ^14^C‐CO_2_ are processed within the plant and nodule. The ^14^C acquisition profiles of nodules and bacteroids (Figure 6) illustrate a time frame consistent with known carbon metabolism steps/stages that include: (1) ^14^C‐CO_2_ fixation; (2) ^14^C‐photosynthate flow from leaves to nodules of perhaps approximately 4 h (= *t*_0_ in Figure 6), followed by (3) metabolism by host nodule enzymes and distribution to the symbiosomes and bacteroids (the interval between 4 and 16 h, Figure 6B). The nodule biochemistry required for incorporation of ^32^Pi into relevant carbon compounds (e.g., biosynthesis of 3‐phosphoglycerate) and presentation to the bacteroid represents an additional step that together is likely much slower than the movement of ^32^Pi into the nodule and to the bacteroid, which occurs within minutes (Figure 2). In addition, metabolites such as 3‐phosphoglycerate have a C:N ratio of 3, which would make it more sensitive for detecting and tracking movement of C to and within the nodule as compared to P in this compound.

Summarising, this study focused on the microsymbiont in the bean symbiosis, combining genetic and functional analysis with plant labelling techniques to better understand the importance of how bacteroid P metabolism differs between indeterminate and determinate legume nodules. The *R. tropici* high affinity P transporter is required for an effective symbiosis in this determinate nodule symbiosis (Figure 1, Hu et al. 2018). Although bacteroid AP is clearly upregulated, it appears to have no discernable role in bacteroid P acquisition as compared to Pi transport. However, elimination of bacteroid AP clearly influences bacteroid and nodule carbon acquisition (Figure 6). As such, further study of bacteroid AP activity is warranted to assess the potential impact on nitrogen fixation.

## Author Contributions

**Lina M. Botero:** methodology, investigation, and review. **Thamir Al‐Niemi:** methodology, investigation, and review. **Timothy R. McDermott:** conceptualization, project administration formal analysis, writing, review and editing.

## Conflicts of Interest

The authors declare no conflicts of interest.

## Data Availability

The data that support the findings of this study are available on request from the corresponding author. The data are not publicly available due to privacy or ethical restrictions.

## References

[emi470220-bib-0001] Al‐Niemi, T. S. , M. L. Kahn , and T. R. McDermott . 1997. “P Metabolism in the Bean‐ *Rhizobium tropici* Symbiosis.” Plant Physiology 113, no. 4: 1233–1242. 12223671 10.1104/pp.113.4.1233 PMC158246

[emi470220-bib-0002] Al‐Niemi, T. S. , M. L. Kahn , and T. R. McDermott . 1998. “Phosphorus Uptake by Bean Nodules.” Plant and Soil 198, no. 1: 71–78.

[emi470220-bib-0003] Botero, L. M. , T. S. Al‐Niemi , and T. R. McDermott . 2000. “Characterization of Two Inducible Phosphate Transport Systems in *Rhizobium tropici* .” Applied and Environmental Microbiology 66, no. 1: 15–22. 10618197 10.1128/aem.66.1.15-22.2000 PMC91779

[emi470220-bib-0004] Cassman, K. , D. Munns , and D. Beck . 1981. “Growth of Rhizobium Strains at Low Concentrations of Phosphate.” Soil Science Society of America Journal 45, no. 3: 520–523.

[emi470220-bib-0005] Cassman, K. , A. Whitney , and R. Fox . 1981. “Phosphorus Requirements of Soybean and Cowpea as Affected by Mode of N Nutrition.” Agronomy Journal 73, no. 1: 17–22.

[emi470220-bib-0006] Deng, S. , J. Elkins , L. Da , L. Botero , and T. McDermott . 2001. “Cloning and Characterization of a Second Acid Phosphatase From *Sinorhizobium meliloti* Strain 104A14.” Archives of Microbiology 176, no. 4: 255–263. 11685369 10.1007/s002030100311

[emi470220-bib-0007] Deng, S. , M. Kahn , and T. McDermott . 1998. “Characterization and Transposon Mutagenesis of a Non‐Specific Acid Phosphatase Cloned From *Rhizobium meliloti* .” Archives of Microbiology 170: 18–26. 9639599 10.1007/s002030050610

[emi470220-bib-0008] Fredeen, A. L. , T. K. Raab , I. M. Rao , and N. Terry . 1990. “Effects of Phosphorus Nutrition on Photosynthesis in *Glycine max* (L.) Merr.” Planta 181, no. 3: 399–405. 24196818 10.1007/BF00195894

[emi470220-bib-0009] Fredeen, A. L. , I. M. Rao , and N. Terry . 1989. “Influence of Phosphorus Nutrition on Growth and Carbon Partitioning in *Glycine max* .” Plant Physiology 89, no. 1: 225–230. 16666518 10.1104/pp.89.1.225 PMC1055823

[emi470220-bib-0010] Goldstein, A. 1992. “Phosphate Starvation Inducible Enzymes and Proteins in Higher Plants.” In Society for Experimental Biology Seminar Series. Cambridge University Press.

[emi470220-bib-0011] Graham, P. H. , and C. P. Vance . 2003. “Legumes: Importance and Constraints to Greater Use.” Plant Physiology 131, no. 3: 872–877. 12644639 10.1104/pp.017004 PMC1540286

[emi470220-bib-0012] Green, R. T. , A. K. East , R. Karunakaran , J. A. Downie , and P. S. Poole . 2019. “Transcriptomic Analysis of *Rhizobium leguminosarum* Bacteroids in Determinate and Indeterminate Nodules.” Microbial Genomics 5, no. 2: e000254. 30777812 10.1099/mgen.0.000254 PMC6421345

[emi470220-bib-0013] Hamburger, D. , E. Rezzonico , J. MacDonald‐Comber Petétot , C. Somerville , and Y. Poirier . 2002. “Identification and Characterization of the Arabidopsis PHO1 Gene Involved in Phosphate Loading to the Xylem.” Plant Cell 14, no. 4: 889–902. 11971143 10.1105/tpc.000745 PMC150690

[emi470220-bib-0014] Hernández, O. , M. Valdés‐López , G. Ramírez , et al. 2009. “Global Changes in the Transcript and Metabolic Profiles During Symbiotic Nitrogen Fixation in Phosphorus‐Stressed Common Bean Plants.” Plant Physiology 151, no. 3: 1221–1238. 19755543 10.1104/pp.109.143842 PMC2773089

[emi470220-bib-0015] Hu, Y. , J. Jiao , L. X. Liu , et al. 2018. “Evidence for Phosphate Starvation of Rhizobia Without Terminal Differentiation in Legume Nodules.” Molecular Plant‐Microbe Interactions 31, no. 10: 1060–1068. 29663866 10.1094/MPMI-02-18-0031-R

[emi470220-bib-0016] Israel, D. W. 1987. “Investigation of the Role of Phosphorus in Symbiotic Dinitrogen Fixation.” Plant Physiology 84, no. 3: 835–840. 16665531 10.1104/pp.84.3.835 PMC1056679

[emi470220-bib-0017] Israel, D. W. 1993. “Symbiotic Dinitrogen Fixation and Host‐Plant Growth During Development of and Recovery From Phosphorus Deficiency.” Physiologia Plantarum 88, no. 2: 294–300.

[emi470220-bib-0018] Itoh, S. 1987. “Characteristics of Phosphorus Uptake of Chickpea in Comparison With Pigeonpea, Soybean, and Maize.” Soil Science and Plant Nutrition 33, no. 3: 417–422.

[emi470220-bib-0019] Jakobsen, I. 1985. “The Role of Phosphorus in Nitrogen Fixation by Young Pea Plants ( *Pisum sativum* ).” Physiologia Plantarum 64, no. 2: 190–196.

[emi470220-bib-0020] Johnson, J. F. , D. L. Allan , C. P. Vance , and G. Weiblen . 1996. “Root Carbon Dioxide Fixation by Phosphorus‐Deficient *Lupinus albus* (Contribution to Organic Acid Exudation by Proteoid Roots).” Plant Physiology 112, no. 1: 19–30. 12226371 10.1104/pp.112.1.19 PMC157919

[emi470220-bib-0021] Johnson, J. F. , C. P. Vance , and D. L. Allan . 1996. “Phosphorus Deficiency in *Lupinus albus* (Altered Lateral Root Development and Enhanced Expression of Phosphoenolpyruvate Carboxylase).” Plant Physiology 112, no. 1: 31–41. 8819319 10.1104/pp.112.1.31 PMC157920

[emi470220-bib-0022] Kang, Y. S. , Z. Shi , B. Bothner , G. Wang , and T. R. McDermott . 2015. “Involvement of the Acr3 and DctA Anti‐Porters in Arsenite Oxidation in *Agrobacterium tumefaciens* 5A.” Environmental Microbiology 17, no. 6: 1950–1962. 24674103 10.1111/1462-2920.12468

[emi470220-bib-0023] Kashyap, D. R. , L. M. Botero , W. L. Franck , D. J. Hassett , and T. R. McDermott . 2006. “Complex Regulation of Arsenite Oxidation in *Agrobacterium tumefaciens* .” Journal of Bacteriology 188, no. 3: 1081–1088. 16428412 10.1128/JB.188.3.1081-1088.2006 PMC1347330

[emi470220-bib-0024] Krol, E. , and A. Becker . 2004. “Global Transcriptional Analysis of the Phosphate Starvation Response in *Sinorhizobium meliloti* Strains 1021 and 2011.” Molecular Genetics and Genomics 272, no. 1: 1–17. 15221452 10.1007/s00438-004-1030-8

[emi470220-bib-0025] McDermott, T. R. 1998. “Phosphate Metabolism in Rhizobium: Issues, Contrasts, and Comparisons.” In Highlights of Nitrogen Fixing Research, edited by E. Martinez and G. Hernandez , 45–48. Plenum Publishing.

[emi470220-bib-0026] McDermott, T. R. , P. H. Graham , and M. L. Ferrey . 1991. “Competitiveness of Indigenous Populations of *Bradyrhizobium japonicum* Serocluster 123 as Determined Using a Root‐Tip Marking Procedure in Growth Pouches.” Plant and Soil 135, no. 2: 245–250.

[emi470220-bib-0027] McDermott, T. R. , and M. L. Kahn . 1992. “Cloning and Mutagenesis of the *Rhizobium meliloti* Isocitrate Dehydrogenase Gene.” Journal of Bacteriology 174, no. 14: 4790–4797. 1320616 10.1128/jb.174.14.4790-4797.1992 PMC206277

[emi470220-bib-0028] McKay, I. A. , and M. A. Djordjevic . 1993. “Production and Excretion of Nod Metabolites by *Rhizobium leguminosarum* Bv. Trifolii Are Disrupted by the Same Environmental Factors That Reduce Nodulation in the Field.” Applied and Environmental Microbiology 59, no. 10: 3385–3392. 16349071 10.1128/aem.59.10.3385-3392.1993 PMC182463

[emi470220-bib-0029] Metcalf, W. W. , P. M. Steed , and B. L. Wanner . 1990. “Identification of Phosphate Starvation‐Inducible Genes in *Escherichia coli* K‐12 by DNA Sequence Analysis of Psi::lacZ(Mu d1) Transcriptional Fusions.” Journal of Bacteriology 172, no. 6: 3191–3200. 2160940 10.1128/jb.172.6.3191-3200.1990 PMC209124

[emi470220-bib-0030] Mimura, T. 1999. “Regulation of Phosphate Transport and Homeostasis in Plant Cells.” In International Review of Cytology, edited by K. W. Jeon , 149–200. Academic Press.

[emi470220-bib-0031] Mullen, M. D. , D. W. Israel , and A. Wollum . 1988. “Effects of Bradyrhizobium Japonicum and Soybean ( *Glycine max* (L.) Merr.) Phosphorus Nutrition on Nodulation and Dinitrogen Fixation.” Applied and Environmental Microbiology 54, no. 10: 2387–2392. 16347750 10.1128/aem.54.10.2387-2392.1988 PMC204268

[emi470220-bib-0032] Nguyen, N. N. T. , J. Clua , P. V. Vetal , et al. 2021. “PHO1 Family Members Transport Phosphate From Infected Nodule Cells to Bacteroids in *Medicago truncatula* .” Plant Physiology 185, no. 1: 196–209. 33631809 10.1093/plphys/kiaa016 PMC8133656

[emi470220-bib-0033] Ormeño‐Orrillo, E. , P. Menna , L. G. P. Almeida , et al. 2012. “Genomic Basis of Broad Host Range and Environmental Adaptability of *Rhizobium tropici* CIAT 899 and Rhizobium sp. PRF 81 Which Are Used in Inoculants for Common Bean ( *Phaseolus vulgaris* L.).” BMC Genomics 13: 735. 23270491 10.1186/1471-2164-13-735 PMC3557214

[emi470220-bib-0034] Pereira, P. , and F. Bliss . 1987. “Nitrogen Fixation and Plant Growth of Common Bean ( *Phaseolus vulgaris* L.) at Different Levels of Phosphorus Availability.” Plant and Soil 104, no. 1: 79–84.

[emi470220-bib-0035] Pereira, P. , and F. Bliss . 1989. “Selection of Common Bean ( *Phaseolus vulgaris* L.) for N2 Fixation at Different Levels of Available Phosphorus Under Field and Environmentally‐Controlled Conditions.” Plant and Soil 115, no. 1: 75–82.

[emi470220-bib-0036] Pongsakul, P. , and E. S. Jensen . 1991. “Dinitrogen Fixation and Soil N Uptake by Soybean as Affected by Phosphorus Availability.” Journal of Plant Nutrition 14, no. 8: 809–823.

[emi470220-bib-0037] Powell, C. L. 1977. “Mycorrhizas in Hill Country Soils: III. Effect of Inoculation on Clover Growth in Unsterile Soils.” New Zealand Journal of Agricultural Research 20, no. 3: 343–348.

[emi470220-bib-0038] Preiss, J. 1984. “Starch, Sucrose Biosynthesis and Partition of Carbon in Plants Are Regulated by Orthophosphate and Triose‐Phosphates.” Trends in Biochemical Sciences 9, no. 1: 24–27.

[emi470220-bib-0039] Qin, L. , J. Zhao , J. Tian , et al. 2012. “The High‐Affinity Phosphate Transporter GmPT5 Regulates Phosphate Transport to Nodules and Nodulation in Soybean.” Plant Physiology 159, no. 4: 1634–1643. 22740613 10.1104/pp.112.199786 PMC3425202

[emi470220-bib-0040] Raghothama, K. 1999. “Phosphate Acquisition.” Annual Review of Plant Biology 50, no. 1: 665–693. 10.1146/annurev.arplant.50.1.665 15012223

[emi470220-bib-0041] Reibach, P. H. , and J. G. Streeter . 1983. “Metabolism of C‐Labeled Photosynthate and Distribution of Enzymes of Glucose Metabolism in Soybean Nodules.” Plant Physiology 72, no. 3: 634–640. 16663058 10.1104/pp.72.3.634 PMC1066293

[emi470220-bib-0042] Ribet, J. , and J. J. Drevon . 1995a. “Increase in Permeability to Oxygen and in Oxygen Uptake of Soybean Nodules Under Limiting Phosphorus Nutrition.” Physiologia Plantarum 94, no. 2: 298–304.

[emi470220-bib-0043] Ribet, J. , and J.‐J. Drevon . 1995b. “Phosphorus Deficiency Increases the Acetylene‐Induced Decline in Nitrogenase Activity in Soybean ( *Glycine max* (L.) Merr.).” Journal of Experimental Botany 46, no. 10: 1479–1486.

[emi470220-bib-0044] Sa, T.‐M. , and D. W. Israel . 1991. “Energy Status and Functioning of Phosphorus‐Deficient Soybean Nodules.” Plant Physiology 97, no. 3: 928–935. 16668533 10.1104/pp.97.3.928 PMC1081106

[emi470220-bib-0045] Santos‐Beneit, F. 2015. “The Pho Regulon: A Huge Regulatory Network in Bacteria.” Frontiers in Microbiology 6: 402. 25983732 10.3389/fmicb.2015.00402 PMC4415409

[emi470220-bib-0046] Schulze, J. 2004. “How Are Nitrogen Fixation Rates Regulated in Legumes?” Journal of Plant Nutrition and Soil Science 167, no. 2: 125–137.

[emi470220-bib-0047] Simon, R. , U. Priefer , and A. Pühler . 1983. “A Broad Host Range Mobilization System for In Vivo Genetic Engineering: Transposon Mutagenesis in Gram Negative Bacteria.” Bio/Technology 1, no. 9: 784–791.

[emi470220-bib-0048] Singleton, P. , H. AbdelMagid , and J. Tavares . 1985. “Effect of Phosphorus on the Effectiveness of Strains of *Rhizobium japonicum* .” Soil Science Society of America Journal 49, no. 3: 613–616.

[emi470220-bib-0049] Smart, J. B. , M. J. Dilworth , and A. D. Robson . 1984. “Effect of Phosphorus Supply on Periplasmic Protein Profiles in Rhizobia.” Archives of Microbiology 140, no. 2: 287–290.

[emi470220-bib-0050] Sulieman, S. , and L. S. Tran . 2015. “Phosphorus Homeostasis in Legume Nodules as an Adaptive Strategy to Phosphorus Deficiency.” Plant Science 239: 36–43. 26398789 10.1016/j.plantsci.2015.06.018

[emi470220-bib-0051] Summers, M. L. , J. G. Elkins , B. A. Elliott , and T. R. McDermott . 1998. “Expression and Regulation of Phosphate Stress Inducible Genes in *Sinorhizobium meliloti* .” Molecular Plant‐Microbe Interactions 11, no. 11: 1094–1101. 9805396 10.1094/MPMI.1998.11.11.1094

[emi470220-bib-0052] Tian, J. , P. Venkatachalam , H. Liao , X. Yan , and K. Raghothama . 2007. “Molecular Cloning and Characterization of Phosphorus Starvation Responsive Genes in Common Bean ( *Phaseolus vulgaris* L.).” Planta 227, no. 1: 151–165. 17701202 10.1007/s00425-007-0603-2

[emi470220-bib-0053] VanBogelen, R. A. , E. R. Olson , B. L. Wanner , and F. C. Neidhardt . 1996. “Global Analysis of Proteins Synthesized During Phosphorus Restriction in *Escherichia coli* .” Journal of Bacteriology 178, no. 15: 4344–4366. 8755861 10.1128/jb.178.15.4344-4366.1996 PMC178200

[emi470220-bib-0054] Vance, C. P. 2001. “Symbiotic Nitrogen Fixation and Phosphorus Acquisition. Plant Nutrition in a World of Declining Renewable Resources.” Plant Physiology 127, no. 2: 390–397. 11598215 PMC1540145

[emi470220-bib-0055] Vance, C. P. , C. Uhde‐Stone , and D. L. Allan . 2003. “Phosphorus Acquisition and Use: Critical Adaptations by Plants for Securing a Nonrenewable Resource.” New Phytologist 157, no. 3: 423–447. 33873400 10.1046/j.1469-8137.2003.00695.x

[emi470220-bib-0056] Wanner, B. L. 1996. “Phosphorus Assimilation and Control of the Phosphate Regulon.” In Escherichia coli and Salmonella *yphimurium* Cellular and Molecular Biology, edited by F. C. Neidharot , R. I. Curtiss , C. A. Gross , et al. American Society for Microbiology.

[emi470220-bib-0057] Yuan, Z. C. , R. Zaheer , and T. M. Finan . 2006. “Regulation and Properties of PstSCAB, a High‐Affinity, High‐Velocity Phosphate Transport System of *Sinorhizobium meliloti* .” Journal of Bacteriology 188, no. 3: 1089–1102. 16428413 10.1128/JB.188.3.1089-1102.2006 PMC1347321

